# Reactive oxygen species-responsive HET0016 prodrug-loaded liposomes attenuate neuroinflammation and improve neurological deficit in a rat model of juvenile traumatic brain injury

**DOI:** 10.3389/fnins.2023.1153349

**Published:** 2023-03-22

**Authors:** Jun Qin, Xiaoli Chen, Rui Wang, Zedan Tian, Yang Li, Shiyu Shu

**Affiliations:** ^1^Department of Anesthesiology, The Second Affiliated Hospital of Chongqing Medical University, Chongqing, China; ^2^College of Pharmacy, Chongqing Medical University, Chongqing, China

**Keywords:** traumatic brain injury, HET0016, neuroinflammation, reactive oxygen species responsive, liposomes

## Abstract

The arachidonic acid pathway metabolite 20-hydroxyeicosatetraenoic acid (20-HETE) contributes to ischemia/reperfusion brain injury. Inhibition of 20-HETE formation can protect the developing brain from global ischemia. In previous studies, we have found that treatment with the 20-HETE synthesis inhibitor N-hydroxy-N-4-butyl-2-methylphenylformamidine (HET0016) can protect the immature brain from traumatic brain injury (TBI), but its hydrophobic nature limits its full potential. We designed a reactive oxygen species-responsive HET0016 prodrug, which consists of a thioketal link between HET0016 and stearyl alcohol (HET-TK-SA), and used the nanoprodrug strategy to successfully synthesize liposomes HET0016 prodrug liposomes (HPLs) to facilitate the application of HET0016 in protection from TBI. HPLs demonstrated spherical shape, size of about 127.8 nm, a zeta potential of −28.8 mv, a narrow particle size distribution and good stability. Male rats at postnatal day 16–17 underwent controlled cortical impact (CCI) followed by intravenous injection with vehicle or HET0016 (1 mg/kg, 2 h post-injury, once/day for 3 days). The results of the *in vivo* demonstrated that HPLs has good biosafety and can pass through the blood-brain barrier. Not only that compared with HET0016, HPLs better-inhibited inflammation and improved neuronal degeneration, which further led to lesion volume reduction, upgraded behavioral task performance, and ameliorated the degree of TBI impairment. Our results demonstrated HPLs could be a new strategy for juvenile TBI therapy.

## 1. Introduction

Traumatic brain injury (TBI) refers to structural brain damage or brain dysfunction caused by external mechanical forces. It is one of the leading causes of disability and death in children and adolescents (Nasr et al., 2019). TBI not only affects brain development resulting in intellectual, motor, and sensory damage but also has long-term effects on learning, living, and social activities for the patient, leading to a significant financial burden on society. Due to their age and potential for development, children typically suffer more negative long-term effects than adults (Cigel et al., 2021). Moreover, the current treatments for pediatric TBI are not satisfactory because most of them are based on the experiences of adults (e.g., nimodipine, progesterone, tirilazad, etc.) (Appavu et al., 2019). Nevertheless, the results are of little reference value because most laboratory studies still use adult animal models and lack human age-matched animal age criteria. Therefore, the study of TBI in immature brains is a research focus and also a challenge in neuroscience.

Our previous study found that N-hydroxy-n-4-butyl-2-methyl phenyl amidine (HET0016), an inhibitor of 20-HETE synthesis, improved acute and long-term histological and functional outcomes after TBI in the developing brains of rats. This improvement was attributed to a reduced neuroinflammatory response due to the inhibition of microglia activation (Shu et al., 2019). Although HET0016 has great potential in the treatment of TBI in the developing brain, its role as a neuroprotective agent is hindered severely because of its poor water solubility, poor distribution *in vivo*, and short half-life (Miyata et al., 2001; Poloyac et al., 2006; Jain et al., 2017). Therefore, there is an urgent need to develop a new and efficient administration strategy for HET0016.

With the development of nanotechnology, nano-formulations provide an alternative strategy for TBI therapy. Compared with traditional formulation, nano-formulation not only ameliorates the hydrophobicity, prolongs the circulation time, and realizes the controlled release properties of drugs but also actively targets drug delivery sites by special surface modification (Jain et al., 2020). Advances in nanotechnology show great potential for treating a range of adult neurological diseases such as cancer, Alzheimer’s disease, and ischemic stroke (Casanova et al., 2019; Saraf et al., 2019). Meanwhile, positive progress has been made in applying nanobiotechnology to brain injuries such as TBI (Wu et al., 2019; Xue et al., 2020), while there is comparatively little study on using nanotechnology to treat brain damage in the developing brain.

Studies have shown that neuroinflammation after TBI leads to blood-brain barrier degradation, edema, metabolic disturbances, microglia activation, and infiltration of peripheral immune cells (Khaksari et al., 2011; Kovesdi et al., 2012; Wallenquist et al., 2012). These immune cells release reactive oxygen species (ROS), which specifically harm the membranes of lipid-rich cells in the neurological system (Uttara et al., 2009). Since ROS are endogenous and highly reactive, ROS-based stimulus-response nanocarriers have broad application and development potential with their advantages of targeted drug delivery and reducing drug toxicity (Kwon et al., 2019). Therefore, by developing suitable ROS-responsive nanocarriers, drug-specific controlled release under ROS activation at the illness site can be achieved, leading to intelligent drug release and accurate treatment.

**FIGURE 1:**
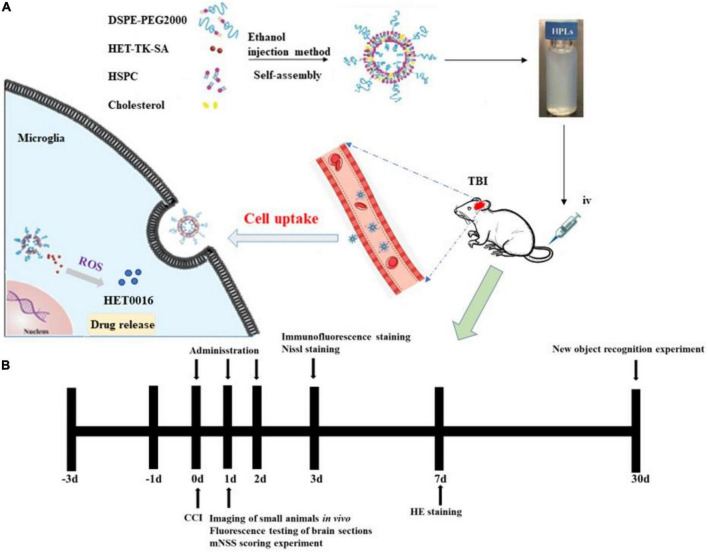
HPLs preparation and protocol schedule. **(A)** Schematic diagram of HET0016 prodrug liposomes (HPLs) from preparation to cellular uptake. **(B)** Experimental protocol and timeline.

In this study, a ROS-responsive HET0016 prodrug with a thioketal link between HET0016 and stearin was synthesized (as described in Figure 1). After TBI, the higher concentration of ROS (Xie et al., 2019) in the injured brain tissue caused the thioketal bond in the prodrug complex to effectively break (Qian et al., 2021) and release the active therapeutic component (HET0016). In subsequent experiments, liposomes of the prodrug complex were prepared and relevant physicochemical properties, such as hydrodynamic size, zeta potential, stability, and drug release behavior, were evaluated in detail. Then, we tried to explore whether the pharmaceutics of HET0016 prodrug liposomes (HPLs) were superior to those of HET0016 alone. The effects of HPLs on brain lesion volume, neuronal damage, microglia activation, and behavioral results were studied in cortical controlled injury (CCI) animal models.

## 2. Materials and methods

### 2.1. Preparation and characterization of HPLs

#### 2.1.1. Synthesis and characterization of the HET0016 prodrug

**FIGURE 2:**
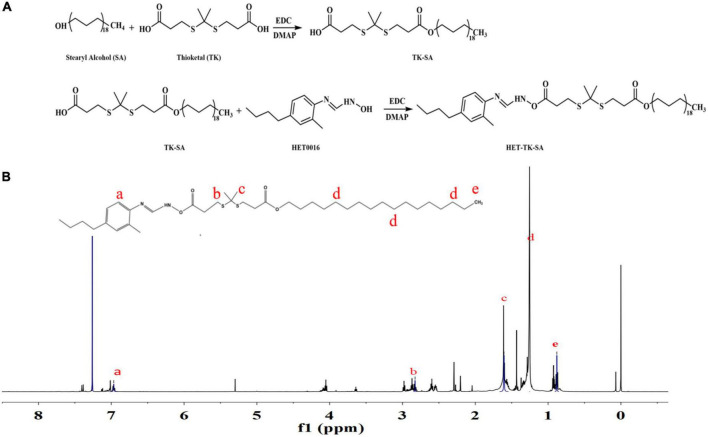
Preparation and structure verification of prodrug. **(A)** The synthesis of HET-TK-SA. **(B)** The ^1^H NMR spectra of HET-TK-SA in DMSO-D6: The characteristic peak of the carboxyl group in HET-TK-SA disappears at 12.01 ppm, the characteristic peak of HET0016 is when the chemical shift appears at 6.97–7.16 ppm, and the characteristic peak of stearyl alcohol appears at 0.88–1.25 ppm, indicating that the carboxyl group successfully reacts with HET0016, and HET-TK-SA is successfully synthesized.

TK-SA was synthesized from thioketal (TK) and stearyl alcohol (SA) by an N1-((ethylimino)methylene)-N3/4-dimethyla-minopyridine (EDC/DMAP; Aladdin Industrial, China) esterification reaction, and then HET0016 (Cayman, USA) was conjugated to it by the same reaction system; the prodrug HET-TK-SA was synthesized, the target product HET-TK-SA obtained by separation purification by silica gel column chromatography, monitored by thin layer analysis, collected by spin evaporation, and the conjugate characterized by 1H nuclear magnetic resonance (^1^H NMR). The synthetic route of the HET0016 prodrug is shown in Figure 2A.

#### 2.1.2. Liposomes preparation and characterization

Prodrug demonstrated inferior *in vitro* stability, and precipitation occurred when they were stored in a refrigerator at 4°C for 48 h. Because of their various unique characteristics, liposomes have been significantly developed both in clinically approved products and new experimental applications compared with other nanotechnology-based drug delivery systems. Therefore, we prepared prodrug-loaded liposomes by the ethanol injection method: in brief, 10 mg of the HET-TK-SA, 100 mg of Hydrogenated Soybean Phosphotidylcholine (HSPC), 25 mg of cholesterol, and 34 mg of DSPE-PEG2000 were co-dissolved in 2.5 ml of anhydrous ethanol to form an oil phase. The oil phase was dispersed into normal saline, and the organic solvent in the emulsion was removed by rotary evaporation after ultrasonic homogenization. Finally, the solution was extruded through a 0.22 mm pore-sized polyether sulfone membrane and stored at 4°C. After proper dilution of the liposomes, the particle size, polydispersity index (PDI), and zeta potential of the nanoparticles were determined by laser particle size measurement, and the morphology was observed by transmission electron microscopy.

### 2.2. Stability studies

HPLs was stored in a covered transparent vial at 4°C for 15 days. Samples were withdrawn periodically and physical stability was assessed by determining any change in physical appearances like a variation of color, sedimentation and aggregation, particle size, size distribution and zeta potential on 0, 3, 7, and 15th day.

### 2.3. *In vitro* drug release study

The liposomes were placed with different components: (1) phosphate buffer solution (PBS, pH 7.4), (2) PBS with 1 mM H_2_O_2_, and the release rate was examined at 100 rpm/min in a constant temperature shaker at 37°C. Aliquots were taken at predetermined time intervals. The content of HET0016 released at the indicated time points was determined by HPLC. The chromatographic conditions and determination of HET0016 were as follows: Agilent C18 column (4. 6 mm × 250 mm, 5 μm); mobile phase ammonium acetate-methanol (80:20); volumetric flow rate 1. 0 mL/min; column temperature 35°C; detection wavelength 256 nm; injection volume 20 μL.

### 2.4. CCI model and drug administration

Male Sprague-Dawley (SD) rats of 16–17 postnatal days (PNDs) were purchased from Dashuo Biotechnology Co., Ltd. (Chengdu, China). They were reared at 20 ± 2°C and 50–60% relative humidity for 12 cycles of light and dark. All the experimental protocols were approved by Chongqing Medical University Ethics Committee for Animal Experimentation following the principles of laboratory and animal care of the University (Protocol 2022-200; Chongqing, China).

Traumatic brain injury was induced by CCI. Rats were anesthetized with pentobarbital sodium (30 mg/kg) and mounted in a stereotaxic frame. After shaving and disinfection, the rats’ skulls underwent craniotomy at the left cortex using a drill. CCI was induced using a 6-mm flat metal impactor tip at 5.5 m/sec, duration of 50 msec, and depth of 1.5 mm (Robertson et al., 2013). The scalp incision was closed with interrupted sutures. Anesthesia was discontinued, and the rats were awakened and returned to the dam with littermates. Sham animals underwent all surgical procedures without sustaining the cortical impact. We randomly divided the rats into four groups: the sham group, TBI + vehicle group, TBI + HET0016 group, and TBI + HPLs group. HET0016 (1 mg/kg) or an equivalent amount of saline was injected into the tail vein of the rats at 2 h after CCI. The drug was administered continuously for 3 days. 24 h after the last administration, the rats were anesthetized with sodium pentobarbital (40 mg/kg in intraperitoneal) followed by transcardial perfusion of 0.9% saline (4°C), followed by 4% paraformaldehyde (4°C). After severing the brain for tissue dehydration and paraffin embedding, the paraffin blocks were trimmed into coronal sections (5 μm) using a microtome for subsequent Nissl staining and immunofluorescence experiments. The remaining batches of rats were selected to be executed at 24 h, 7 days, and 30 days after TBI for relevant experiments (Figure 1B).

### 2.5. Targeting and biological safety evaluation *in vivo*

*In vivo* imaging was used to investigate the penetration of HPLs through the blood-brain barrier. Liposomes containing the same amount of DiR were prepared by the same method. Liposomes were injected intravenously to observe the distribution of drug.

The rat model of TBI was established, and the rats were divided into two groups. The rats were injected with liposomes and normal saline in the tail vein for 7 days and then euthanized. The tissues of the heart, liver, lung, spleen, and kidney were taken and prepared into paraffin sections after dehydration. The physiological toxicity of liposomes to TBI rats was investigated by hematoxylin-eosin staining (HE staining).

### 2.6. Lesion volume and neuronal injury assessment

We assessed lesion volumes 3 days after TBI. Experiments were performed using toluidine blue to stain 5-μm coronal sections of the brain and Image J software to determine the area of defects in each group of sections. Lesion volume was assessed following a previous report but with some modifications (Dash et al., 2010).


B⁢r⁢a⁢i⁢n⁢i⁢n⁢j⁢u⁢r⁢y⁢v⁢o⁢l⁢u⁢m⁢e=Σ⁢l⁢a⁢y⁢e⁢r⁢t⁢h⁢i⁢c⁢k⁢n⁢e⁢s⁢s×S⁢(a⁢r⁢e⁢a⁢o⁢f⁢i⁢n⁢j⁢u⁢r⁢y)

After the paraffin sections were prepared for Nissl staining, cortical and hippocampal neurons on the damaged side were observed under Olympus microscope (Tokyo, Japan), and the number of surviving neurons in the cerebral cortex and hippocampal CA1 area was counted (×400). For cortical counting, the counting area of interest (ROI) was randomly selected in a region 200 μm away from the edge of the trauma lesion, and 3 independent and non-repetitive visual fields were counted under a field of 400 magnification, and their average values were taken as the number of surviving neurons in the section. For the CAI region of the hippocampus, the same ROI part was selected in the hippocampus for counting. CAI area is the part of the dorsal hippocampus containing small pyramidal neurons, and a fixed length was selected from the starting part of the CAI region for counting.

### 2.7. Microglia activation observation with immunofluorescence staining

Microglia were characterized activation by co-labeling for Iba-1 and CD68 receptor, a marker of the proinflammatory profile in microglia (Allen et al., 2019). The brain slices were blocked with 5% bovine serum at room temperature for 1 h, and the staining for Iba-1 and CD68 was performed. The sections were incubated with primary antibodies at 4°C overnight. After washing 5 times with PBST (PBS + 0.1% Tween 20) for 10 min, sections were incubated with secondary antibodies for 1 h at room temperature and washed 3 times with PBST. After final washing, the nucleus was stained with an antifading mounting medium containing DAPI. The sections were then observed under a confocal microscope (Leica, TCS SP8).

### 2.8. Assessment of neurological function with mNSS

Neurological function evaluation was performed 24 h after TBI using modified neurological severity scores (mNSS), which is a composite test including motor, sensory, and reflex tests (Xu et al., 2017). Briefly, the mNSS test is graded on a scale of 0–18 scores; a score of 0 indicates a normal neurological function and 18 is the maximal deficit score. The higher the score, the more severe the brain injury is. All neurobehavioral tests were carried out by two investigators who were blinded to the experimental groups.

### 2.9. Assessment of learning and memorial function with novel objective recognition

Thirty days after CCI, a researcher conducted a blinded test on the test group. On day 1, rats were placed individually in an empty 0.4 × 0.4 m plastic box for 5 min to acclimatize to the chamber environment. On day 2, the rats were placed in the same box with 2 objects of the same shape and color for 5 min for familiarity and memory coding. Twenty-four hours later, the mice were placed in the box again, but a familiar object was replaced with a new object of a different color and shape. The box and objects were cleaned with 70% alcohol before and after each behavioral evaluation to avoid any olfactory cues. The time spent exploring was recorded in the familiarization and testing sessions. Videos were coded and scored by the investigator blinded to the treatments. The data collected were analyzed using EthoVision XT V.13 video-tracking software (Leesburg, VA, USA). During video playback, the time spent interacting with the new and familiar objects was analyzed for each rat. The discrimination index (DI) was used to assess the learning and memory ability of the animals.


D⁢I=(N-F)/(N+F)×100%

N (new) is the time spent exploring new objects and F (familiar) is the time spent exploring familiar objects (Deselms et al., 2016).

### 2.10. Statistical analysis

SPSS 18.0 software (SPSS Inc., Chicago, IL, USA) was used for statistical analysis. Statistical significance was verified using one-way analysis of variance (ANOVA) followed by Tukey’s test for multiple comparisons. Differences were considered statistically significant at a value of *p* < 0.05.

## 3. Results

### 3.1. Preparation and characterization of HPLs

#### 3.1.1. Synthesis and identification of prodrug

The results of ^1^H NMR (Figure 2B) indicated that a ROS-responsive prodrug with a thioketal link between HET0016 and SA was successfully synthesized.

#### 3.1.2. Preparation and characterization of liposomes

**FIGURE 3:**
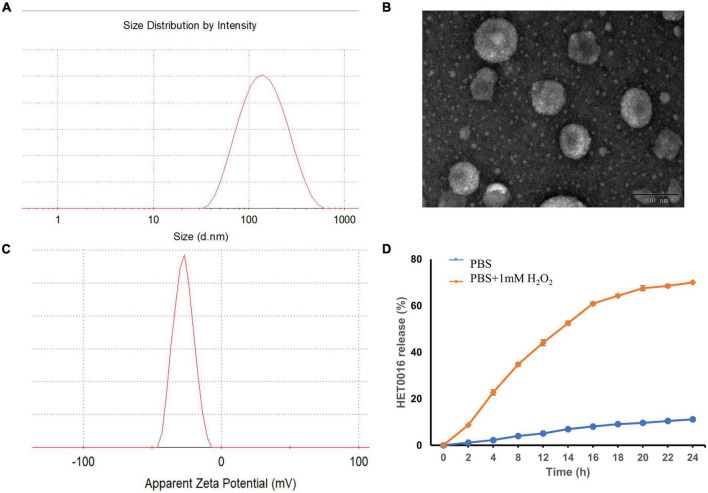
The characterization of HPLs. **(A)** The particle size distribution. **(B)** TEM image of the HPLs, scale bar = 100 nm. **(C)** The particle zeta distribution. **(D)** *In vitro* release profiles of HET0016 from HPLs in PBS (pH 7.4) and PBS (pH 7.4) with 1 mM H_2_O_2_. Experiments were performed in triplicate and value were mean ± SD.

The mean diameter and zeta potential of HPLs were 126.77 ± 1.84 nm and − 18.2 ± 0.89 mV, respectively. The results of DLS and TEM are shown in Figure 3, which indicated that the prepared HPLs exhibited a uniform and spherical morphology.

Evaluation of the stability of a formulation is important when developing the formulation as a commercial product. The stability of HPLs was assured over a period of 15 days at 4°C by determining physical appearances like a change in color, sedimentation, aggregation, particle size, and PDI. Results showed no change in color, sedimentation, and aggregation in the Liposomes over a period of 15 days. In addition, no statistically significant change in particle size and PDI was observed, which indicated that the HPLs showed good stability (Table 1).

**TABLE 1:** Physical stability parameters for HPLs.

	Change of color/sedimentation/aggregation	Particle size [avg (nm)]	PDI	Zeta potential (mV)
At 0 day	Not observed	126.77 ± 1.84	0.205 ± 0.009	− 18.2 ± 0.89
At 7 day	Not observed	128.82 ± 3.23	0.225 ± 0.007	− 17.6 ± 0.58
At 15 day	Not observed	132.97 ± 4.13	0.241 ± 0.002	− 16.8 ± 0.44

Data were presented as mean ± SD; *n* = 3. PDI, polydispersity index.

### 3.2. *In vitro* release study

Reactive oxygen species-triggered HPLs release of HET0016 was studied. The cumulative release percentage is shown in Figure 3D. The results show that in PBS containing H_2_O_2_, the cumulative release after 24 h was about 70%. The release of HET0016 was less than 12%, indicating that HPLs had good stability in PBS with a pH value of 7.4.

### 3.3. HPLs have good targeting and biosafety in TBI models

**FIGURE 4:**
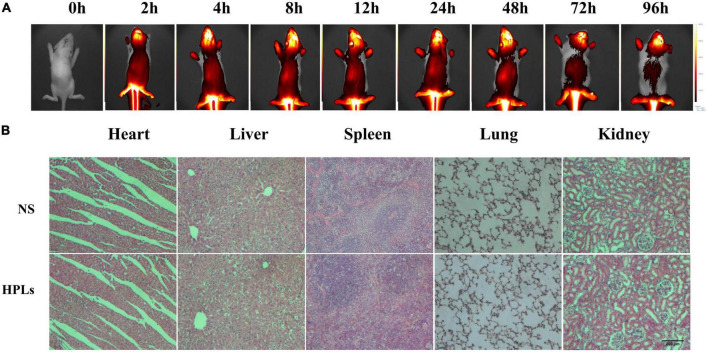
HPLs have good targeting and biosafety in TBI models. **(A)** Fluorescence distribution in rats of different time point after injection of DiR-LIP **(B)** Histological examination of the heart, liver, spleen, lung, and kidney samples excised from CCI rats on the 7th day after the respective treatment with saline and HPLs. The images were observed under a microscope at a magnification of 400 times.

As shown in Figure 4A, the brain showed strong fluorescence signal 2–96 h after injection. The results showed that liposomes had good blood-brain barrier penetration and formed aggregation in the brain. Long-term retention was conducive to drug release and effect.

Figure 4B shows that after intravenous injection of a therapeutic dose of liposomes for 7 days, no obvious pathological changes were found in the HE staining sections of various organs of rats, indicating that the concentration of liposomes in this study had no obvious toxicity and side effects on rats, and the liposomes had good biocompatibility *in vivo*.

### 3.4. HPLs reduced the lesion volume and neuronal degeneration after TBI

**FIGURE 5:**
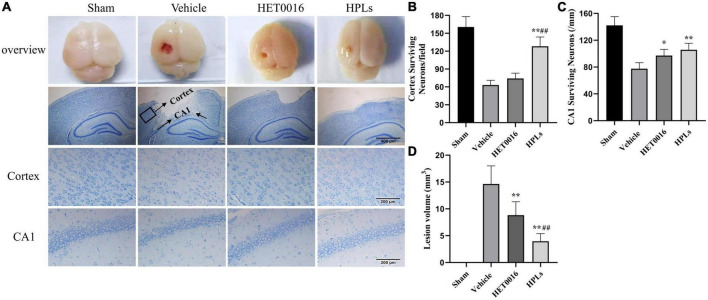
HPLs reduced the lesion volume and neuronal degeneration after TBI. **(A)** Brain defects of rats (magnification of region: 200 times) and Nissl stain in each group (magnification of region: 400 times). Quantitative analysis of the Nissl positive cells in the cortex **(B)** and hippocampus **(C)** on the injured side. **(D)** Quantitative analysis of brain injury volume in each group. Values were mean ± SD. *p < 0.05, **p < 0.01 versus Vehicle, ^#^*p* < 0.05, ^##^*p* < 0.01 versus HET0016, *n* = 6 in each group.

In the sham group, no definite brain tissue defects were found after Nissl staining (Figures 5A, D), but significant brain tissue defects (14.64 ± 2.84 mm^3^) were seen in the Vehicle group. The treatments with HET0016 and HPLs administered intravenously did not completely prevent the occurrence of brain tissue defects, but they significantly reduced the brain tissue defects caused by this brain trauma (8.83 ± 2.11 mm^3^ in the HET0016 group, 3.96 ± 1.20 mm^3^ in the HPLs group, *p* < 0.05 versus Vehicle group), and the difference was statistically significant, showing an overall protective effect on brain tissue structures.

In the Vehicle group, the numbers of neurons around the cortical contusion region (63 ± 7.46 versus 160.5 ± 15.97 in the sham group) and in the hippocampal CAI area (77.5 ± 8.34 versus 142.17 ± 11.91 in the sham group) were significantly reduced (Figures 5B, C), and the normal form of most neurons disappeared. However, compared with the Vehicle group, the HPLs group had significantly reduced abnormal neuronal loss and morphological changes in the cortical (128.17 ± 14.25) and in the hippocampal CAI areas (106.83 ± 8.71, *p* < 0.01 versus Vehicle group), and the recovery effect on abnormal neurons in the cortical area of the HET0016 group was not obvious.

### 3.5. HPLs reduced microglial activation after TBI

**FIGURE 6:**
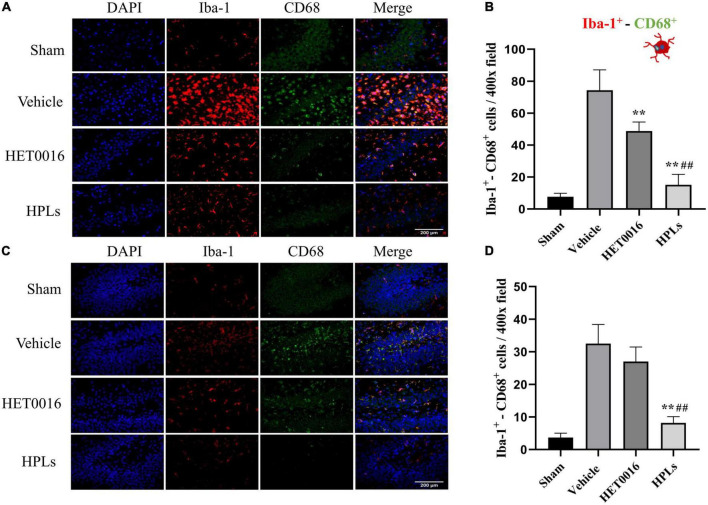
HPLs reduced microglial activation after TBI. The Iba-1^+^- CD68^+^ stain in the injured cortical **(A)** area and hippocampus **(C)** of the injured side. The green fluorescence represents the CD 68-positive cells, the red fluorescence represents the Iba-1 cells, and the blue fluorescence represents DAPI (magnification of region: 400 times). **(B)** The quantitative analysis of the Iba-1^+^- CD68^+^ positive cells in groups (in the injured cortical area). **(D)** The quantitative analysis of the Iba-1^+^- CD68^+^ positive cells in groups (hippocampus of the injured side). Values were mean ± SD. **p < 0.01 versus Vehicle, ^##^*p* < 0.01 versus HET0016, *n* = 6 in each group.

As shown in the figures (Figures 6A, B), the number of Iba-1^+^- CD68^+^ microglia was significantly increased in the cerebral cortex of the Vehicle group compared to that of the control group (74.33 ± 11.64 versus 7.67 ± 1.97 in the control group, *p* < 0.01); the HET0016 group (48.83 ± 6.26) and the HPLs group (15.17 ± 5.98) had a significant reduction in the number of Iba-1^+^- CD68^+^ microglia compared to the Vehicle group (*p* < 0.01 versus Vehicle group). In the rat hippocampal region (Figures 6C, D), the number of Iba-1^+^- CD68^+^ microglia was significantly reduced in the HPLs group compared to the Vehicle group (8.17 ± 1.77 versus 32.50 ± 5.35 in the Vehicle group, *p* < 0.01). However, no significant difference was observed between the HET0016 and Vehicle groups (27.00 ± 1.77 versus 32.50 ± 5.35, *p* > 0.05).

### 3.6. HPLs ameliorated TBI-induced deficits in neurological function

**FIGURE 7:**
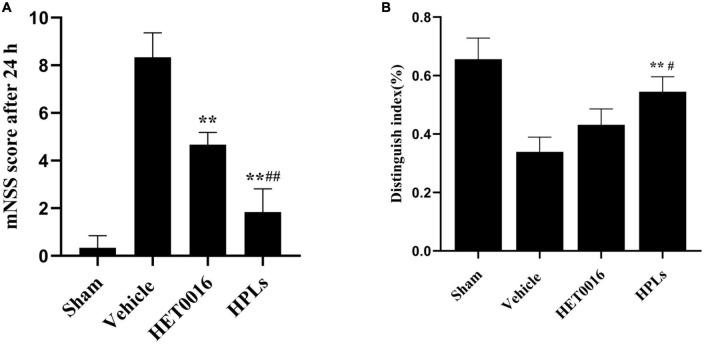
HPLs ameliorated TBI-induced learning/memorial function. **(A)** Neurological function was analyzed using mNSS at 24 h after TBI. **(B)** Analysis using the New Object Recognition Test at 30 day after TBI. Data are presented as mean ± SD. *p < 0.05, **p < 0.01 versus Vehicle, ^#^*p* < 0.05, ^##^*p* < 0.01 versus HET0016, *n* = 6 in each group.

To determine the effect of therapeutic drugs on neurological deficit after TBI, we performed the mNSS test before the rats in each group were euthanized. At 24 h after TBI induction, the scores of the rats in each treatment group were higher than those in the sham group. Compared with the Vehicle group, the neurological function scores of the HET0016 and HPLs groups were significantly lower (*p* < 0.01). There was no significant difference between the HET0016 group and the HPLs group (Figure 7A).

### 3.7. HPLs ameliorated TBI-induced learning/Memorial function

Studies have demonstrated that rats have an innate propensity to explore new objects, and therefore, rats with unimpaired cognitive function use more time to explore new objects in their surroundings, while their exploration time of familiar objects in their surroundings is relatively shorter. The New Object Recognition Test System is a cognitive function test method that exploits the principle that animals have an innate tendency to explore new objects. The discrimination index of old and new objects in the Vehicle group was significantly lower than that in the sham group, while the discrimination index of old and new objects in the HET0016 group and the HPLs group were improved after TBI. The effect was more significant in the HPLs group, and the difference was statistically significant (*p* < 0.05) (Figure 7B).

## 4. Discussion

In this study, HPLs with the appropriate particle size, uniform distribution, slow-release characteristics, high stability, and biosafety were successfully synthesized. Meanwhile, HPLs significantly reduced the volume of brain defects, inhibited microglia activation, alleviated neuronal damage, and improved neurological function scores and long-term behaviors in TBI rats. Liposomes prepared by caudal vein injection can enrich in the focal site of TBI rats and maintain a certain duration of action. Taken together, HPLs can be considered a promising and potential agent for the treatment of TBI in the immature brain by inhibiting neuroinflammation.

The response of the developing brain to traumatic injury is different from the response of the mature, adult brain. There are critical developmental trajectories in the young brain, whereby injury can lead to long term functional abnormalities. The morphology and function of microglia are relatively stable in the mature brain, but in the immature brain, microglia are formed in parallel with neurons, and they are not only related to the health and survival of neurons but also closely related to the formation of synapses, the construction of neuronal networks, and the removal of cell fragments. Therefore, microglia play a key role in the regulation of brain development and brain balance (Nayak et al., 2014). When the immature brain faces TBI, the activation of microglia will destroy the brain’s homeostasis. Abnormal neuronal and synaptic development changes the cell environment and leads to the formation of abnormal neural circuits, which often results in more severe post-traumatic memory deficits and functional damage in immature brains than in mature ones with TBI. As a result, the recovery period may be delayed (Zamani et al., 2020). Therefore, early inhibition of excessive microglia activation is the key to the survival of neurons, the maintenance of intracerebral homeostasis, and the mitigation of secondary injury in immature brains.

Currently, the medical management of traumatic brain injury is only symptomatic. It mainly includes the treatment of intracranial hypertension and epileptic persistent state, as well as the maintenance and cessation of sedation and analgesia of patients (Geeraerts et al., 2018). To date, there are no therapeutic strategies available to prevent the onset and spread of secondary lesions in humans. In adult animal models of TBI, progesterone has been found to have anti-edema properties, reduce lesion volume, and alleviate neurological symptoms. However, Mannix et al. (2014) found that progesterone treatment had no effect on the lesion volume or spatial memory ability of CCI 4-week-old mice, and even resulted in deterioration of motor function in female mice. Previous studies have shown that nicotinamide (NAM vitamin B3) significantly reduces behavioral impairment and improves function in adult animal models (Hoane et al., 2008), whereas Smith et al. (2019) found that nicotinamide treatment in juvenile animals (28 days) did not result in enhanced functional recovery on any behavioral tasks. Melatonin has strong antioxidant and anti-apoptotic effects and is recommended as part of the pediatric TBI management plan (Santini et al., 2018). Although melatonin has significantly improved neural, cognitive, and motor functions in preclinical animal models (Barlow et al., 2019), its efficacy in the treatment of pediatric TBI patients remains controversial. In a randomized, double-blind study, melatonin reduced ADHD in pediatric patients but did not improve overall outcomes (Barlow et al., 2020). In a neonatal brain damage model, Chhor’s research examined the effects of minocycline, a second-generation tetracycline antibiotic with anti-inflammatory properties. Minocycline inhibition of microglia activation reduced the severity of injury 1 day post-injury and reduced ventricular dilation and cell death. However, in this model, minocycline appears to have only a transient neuroprotective effect, as it does not affect injury severity at 5 days post-injury (Chhor et al., 2017). Although some of these medicines have shown neuroprotective effects in animal models, none have been successful in clinical trials, probably due to the strict treatment time window and the heterogeneity of TBI subtypes. At the same time, it is important to note that age-at-injury affects not only the outcome of TBI, but also the pharmacological action of the drug (Delage et al., 2021). However, inflammatory, neurological, and functional consequences after TBI have mainly been studied in experimental adult animal models. Therefore, using new delivery tools in future experimental studies to design therapies that target neuroinflammation at appropriate time points to promote long-term recovery and promote normal brain maturation will be key for the treatment of immature brain TBI.

HET0016 has demonstrated therapeutic efficacy in animal models for the treatment of stroke (Renic et al., 2009; Han et al., 2019), cerebral ischemia-reperfusion (Yang et al., 2012; Zhu et al., 2015), and oxygen-glucose deprivation reoxygenation (Zhang et al., 2017). Recently, the application of HET0016 to immature brain TBI models has also shown positive efficacy. In our earlier study, we found that HET0016 could significantly reduce the activation of microglia, reduce the volume of brain damage at 3 and 30 days after TBI, and enhance motor impairment and memory function in young SD rats after TBI (Shu et al., 2019). Although HET0016 has a good deal of potential for treating the immature brain, its small molecular weight and widespread distribution in the body after administration mean that some of it is broken down and destroyed before it can reach the brain tissue, altering its physicochemical properties and preventing high-quality and quantity delivery to the damaged tissue. In addition, HET0016 has poor absorption, fast metabolism, a short half-life, low bioavailability, and poor efficacy, which greatly limit its drug delivery route and clinical application. Given the shortcomings of HET0016, it is necessary to modify it or to use nano preparation (nanotechnology) to improve its efficacy in TBI.

A prodrug is a chemically altered version of a drug that exhibits little or no biological activity *in vitro*. After entering the body, the substance will undergo biotransformation or metabolism to become a substance with pharmacological activity (Kratz et al., 2008). Prodrug application is used primarily to promote drug targeting and boost bioavailability (Zhigaltsev et al., 2010). Nanodrugs have the advantage of increasing the stability of the drug (Uhrich et al., 1999) and improving the circulation time and distribution behavior of the drug in the body (Fang et al., 2006). Combining the advantages of prodrugs and nanodrugs, we prepared the prodrug complex liposomes HPLs with HET0016 as the model drug, stearyl alcohol as the carrier, and thioketal as the connecting bond. ROS-sensitive prodrugs change the distribution of HET0016 *in vivo* and drug solubility. The hydroxyl functional group of HET0016 is esterified when it is linked with TK, which can protect HET0016 from premature degradation. In addition, the nanodrug can change the tissue distribution of the drug and improve the drug delivery efficiency. Polyethylene glycol (PEG) is added to the surface of liposomes to form a layer that protects the carrier from plasma protein binding (Milla et al., 2012; Xu et al., 2019) and prolongs its circulation time *in vivo*. The combination of these two techniques reduces the degradation of HET0016 during circulation, passively targets the damaged site through the EPR effect, and selectively releases the active ingredient under the influence of the microenvironment. This targeted enrichment in brain tissue creates a prerequisite for liposomes to improve the prognosis of TBI, which is a safe and effective potential treatment for TBI.

There are several potential limitations of this study. First, the pharmacokinetics of HET0016 and the mean residence time following intravenous administration of HPLs were not specifically investigated. Secondly, this study used new object recognition 30 days after TBI to examine the long-term behavioral prognosis of TBI rats; however, new object recognition cognitive outcomes that are partially dependent on hippocampal function may not necessarily translate to the human brain where the hippocampus is more ventrally located. Moreover, the experiments lacked behavioral prognostic results for more distant periods (adult and even old age in rats). Therefore, in future studies, different behavioral experiments will be used to observe the more distant behavioral prognosis of rats at 30 days or even 60 days after TBI. Finally, since significant sex-based outcome changes have been observed in both preclinical models and clinical settings (Smith et al., 2016; Wood et al., 2020), male individuals may exhibit lower resistance to injury compared to female individuals (Du et al., 2004; Demarest and McCarthy, 2015). Therefore, we expect to add the variable of animal sex in subsequent experiments to assess sex differences in response to HET0016 drug treatment.

## 5. Conclusion

In conclusion, this research prepared HPLs with reactive oxygen species responsiveness, which have good solution dispersion and slow-release characteristics, improve the concentration of drugs in the lesions, and produce better therapeutic effects. Overall, using nanoliposomes to deliver HET0016 has the potential to target neuroinflammation and improve its efficacy in the treatment of immature brain TBI.

## Data availability statement

The original contributions presented in this study are included in the article/supplementary material, further inquiries can be directed to the corresponding author.

## Ethics statement

This animal study was reviewed and approved by the Ethics Committee of the Second Affiliated Hospital of Chongqing Medical University.

## Author contributions

JQ, SS, and YL designed the research, participated in discussion, and wrote and revised the manuscript. JQ, XC, ZT, and RW conducted the experiments. All authors contributed to the article and approved the submitted version.
